# Artificial intelligence as a teaching tool for gynaecological ultrasound: A systematic search and scoping review

**DOI:** 10.1002/ajum.12368

**Published:** 2023-11-20

**Authors:** Alison Deslandes, Jodie Avery, Hsiang‐Ting Chen, Mathew Leonardi, George Condous, M. Louise Hull

**Affiliations:** ^1^ Robinson Research Institute University of Adelaide Adelaide South Australia Australia; ^2^ School of Computer and Mathematical Sciences University of Adelaide Adelaide South Australia Australia; ^3^ Department of Obstetrics and Gynecology McMaster University Hamilton Ontario Canada

## Abstract

**Purpose:**

The aim of this study was to investigate the current application of artificial intelligence (AI) tools in the teaching of ultrasound skills as they pertain to gynaecological ultrasound.

**Methods:**

A scoping review was performed. Eight databases (MEDLINE, EMBASE, EMCARE, CINAHL, Scopus, Web of Science, IEEE Xplore and ACM digital library) were searched in December 2022 using predefined keywords. All types of publications were eligible for inclusion so long as they reported the use of an AI tool, included reference to or discussion of teaching or the improvement of ultrasound skills and pertained to gynaecological ultrasound. Conference abstracts and non‐English language papers which could not be adequately translated into English were excluded.

**Results:**

The initial database search returned 481 articles. After screening against our inclusion and exclusion criteria, two were deemed to meet the inclusion criteria. Neither of the articles included reported original research (one systematic review and one review article). Neither of the included articles explicitly provided details of specific tools developed for the teaching of ultrasound skills for gynaecological imaging but highlighted similar applications within the field of obstetrics which could potentially be expanded.

**Conclusion:**

Artificial intelligence can potentially assist in the training of sonographers and other ultrasound operators, including in the field of gynaecological ultrasound. This scoping review revealed however that to date, no original research has been published reporting the use or development of such a tool specifically for gynaecological ultrasound.

## Introduction

Artificial intelligence (AI) is playing an ever‐expanding role in the field of medical imaging, with many systems commercially available and approved for clinical use, which can assist with workflow efficiency, quality assurance of images, image interpretation and clinical decision making.^1^ In the field of gynaecology, transvaginal ultrasound (TVUS) is the first line imaging method for diagnosis and monitoring of pathology. Despite the widespread use of ultrasound in gynaecology, AI in this setting is still immature.^1^, ^2^ Irrespective of this, there is great potential for AI in gynaecological ultrasound to assist with time‐consuming tasks (such as obtaining measurements), providing quality assurance of images and assisting clinicians with decision making.^2^

TVUS is typically widely accessible, especially in high‐resourced healthcare settings. Despite its wide availability, the quality of the imaging obtained is heavily dependent on the skill, experience and expertise of the sonographer which varies greatly from centre to centre.^3^, ^4^, ^5^ Training of sonographers is a labour intensive and costly process, typically involving the need to perform supervised scans with a sufficiently experienced mentor until competence is reached.^6^ With a shortage of qualified sonographers in many locations, and increasing demand for ultrasound, access to training is becoming increasingly challenging due to time pressures on experienced sonographers to perform scans, rather than train beginners.^6^ When upskilling sonographers to perform more advanced techniques, such as endometriosis TVUS and 3D/4D ultrasound,^7^ this challenge is enhanced by a limited number of people currently possessing the skills required to effectively educate others.^8^

Several AI systems have been developed for other ultrasound applications, such as obstetrics and echocardiography, which guide novice users to manipulate the probe and identify correct scanning planes. This then allows for the acquisition of ultrasound data from which basic measurements can be performed, all with little or no formal training in ultrasound scanning.^9^, ^10^ Some systems are also able to provide feedback to operators as to the quality of the image they are obtaining and, in some cases, provide feedback to improve the image in real time.^10^, ^11^, ^12^ Whilst none of these systems replace supervised scanning by becoming as proficient as a diagnostic ultrasound specialist, it is likely that AI holds potential to play a future role in the teaching of certain skills required to perform TVUS. If such tools could be utilised, this may have the potential to close some of the skill gaps related to TVUS and expand access to higher quality ultrasound. As such, the aim of this review was to investigate the current application of AI tools in the teaching of ultrasound skills as they pertain to gynaecological ultrasound.

## Methods

### Study design

An initial search of Google Scholar was performed to assess whether any reviews on this topic had been performed. This review was then planned using the Preferred Reporting Items for Systematic Reviews and Meta‐Analyses extension for Scoping Reviews (PRISMA‐ScR) (Appendix S1)^13^ and the Joanna Briggs Institute (JBI) best practice guidance and reporting items for the development of scoping review protocols.^14^ All methods for inclusion and exclusion, screening and data extraction were planned in advance. The review protocol was published online on Open Science Framework (https://osf.io/ymak4/) on 12 December 2022. Ethics approval for this study was not required as it was drawing upon only previously published, publicly avaliable data.

### Data sources

Assistance was sought from an academic librarian at the University of Adelaide in the development of keywords and search terms. Artificial intelligence was defined as the simulation of human activities by machines or computers. Teaching was defined as training, instruction or tuition to perform a skill. From this, a participant, comparator, context (PCC) plan was created (Table 1). A PCC was selected rather than a Population, Intervention, Comparison, Outcome (PICO) plan as no relevant intervention was being assessed. This PCC informed the keywords ‘Artificial Intelligence’, ‘ultrasound’, ‘gynaecology’ and ‘teaching’. Eight databases were used for the primary search; MEDLINE, EMBASE, EMCARE, CINAHL, Scopus, Web of Science, IEEE Xplore and ACM digital library using the predefined keywords and their synonyms with the appropriate Boolean operators for each. An example of the search strategy used can be seen in Table 2 with a full list of these terms can be seen in Appendix S2. A preliminary search was performed to ensure these terms resulted in some literature being returned and confirm these search terms did not need to be expanded. Papers returned from inception to December 2022 were included and pearling of the returned articles reference lists was performed to identify any other relevant articles.

**Table 1:** The participant, comparator, context (PCC) plan formulated to inform this scoping review.

Participants	Concept	Context
N/A	Artificial intelligence tools	Gynaecological ultrasound
	Teaching	

**Table 2:** The search strategy employed for the Medline database.

AI tools	Ultrasound	Gynaecology	Teaching
Exp Artificial intelligence OR (artificial intelligence OR deep learning OR AI OR machine learning OR computer aided OR computer vision OR Neural Network).ti,ab	Ultrasonography.sh OR (ultraso* OR sonogr* OR POCUS).ti,ab	Gynecology.sh OR (gyn?ecolog* OR female pelvi*).ti,ab	Exp education OR (teach* OR train* OR instruct* OR upskill* OR learn* OR assist*).ti,ab

### Inclusion/exclusion criteria

All types of publications were considered eligible for inclusion in this review including, but not limited to, primary research studies (both quantitative and qualitative design), systematic reviews and meta‐analysis, case reports, protocols, opinion articles and unpublished grey literature.

To be considered for inclusion, articles must have reported the use of an artificial intelligence tool, include reference to or discussion of teaching or the improvement of ultrasound skills and pertain to gynaecological ultrasound. Conference abstracts and non‐English language papers which could not be adequately translated into English were excluded.

### Study selection

All returned articles were saved and imported into Zotero reference managing software (Zotero 6.0.18 for Mac, Corporation for Digital Scholarship, George Mason University, Virginia, USA) and the duplicates removed. The reference list was then imported into the Covidence systematic review management system (Covidence systematic review software, Veritas Health Innovation, Melbourne, Vic., Australia). All articles underwent title and abstract screening by two investigators (AD and JA) to determine relevance against the inclusion and exclusion criteria. Articles not meeting the inclusion criteria were excluded. All remaining articles underwent full text review by two investigators (AD and JA or MLH). If agreement was not reached between the two reviewers as to whether an article is suitable for inclusion, a third investigator (ML) performed a full‐text review to determine suitability for inclusion/exclusion. Studies were not appraised for quality as the purpose of this review was to map current data in line with accepted systematic scoping review methodology.^14^

### Data extraction

Data were extracted by the principal investigator (AD) with assistance from an AI subject matter expert (HTC), into an Excel spreadsheet (Microsoft® Excel for Mac, Version 16.30, Microsoft Corporation, Redmond, Washington, USA). The data extracted included general study details (Author(s), year of publication, country, journal), study type (*e.g*. primary research, systematic review, opinion) and other relevant data were reported such as type of AI tool reported (*e.g*. Machine learning, neural networks, deep learning), details of AI tool development, teaching mechanism employed, aim, methods, results, limitations and conclusions. In one study,^15^ the corresponding author was contacted *via* email (on 02 February 2023 and again on 23 March 2023) to provide further detail on their article; however, this was unsuccessful.

## Results

**Figure 1:**
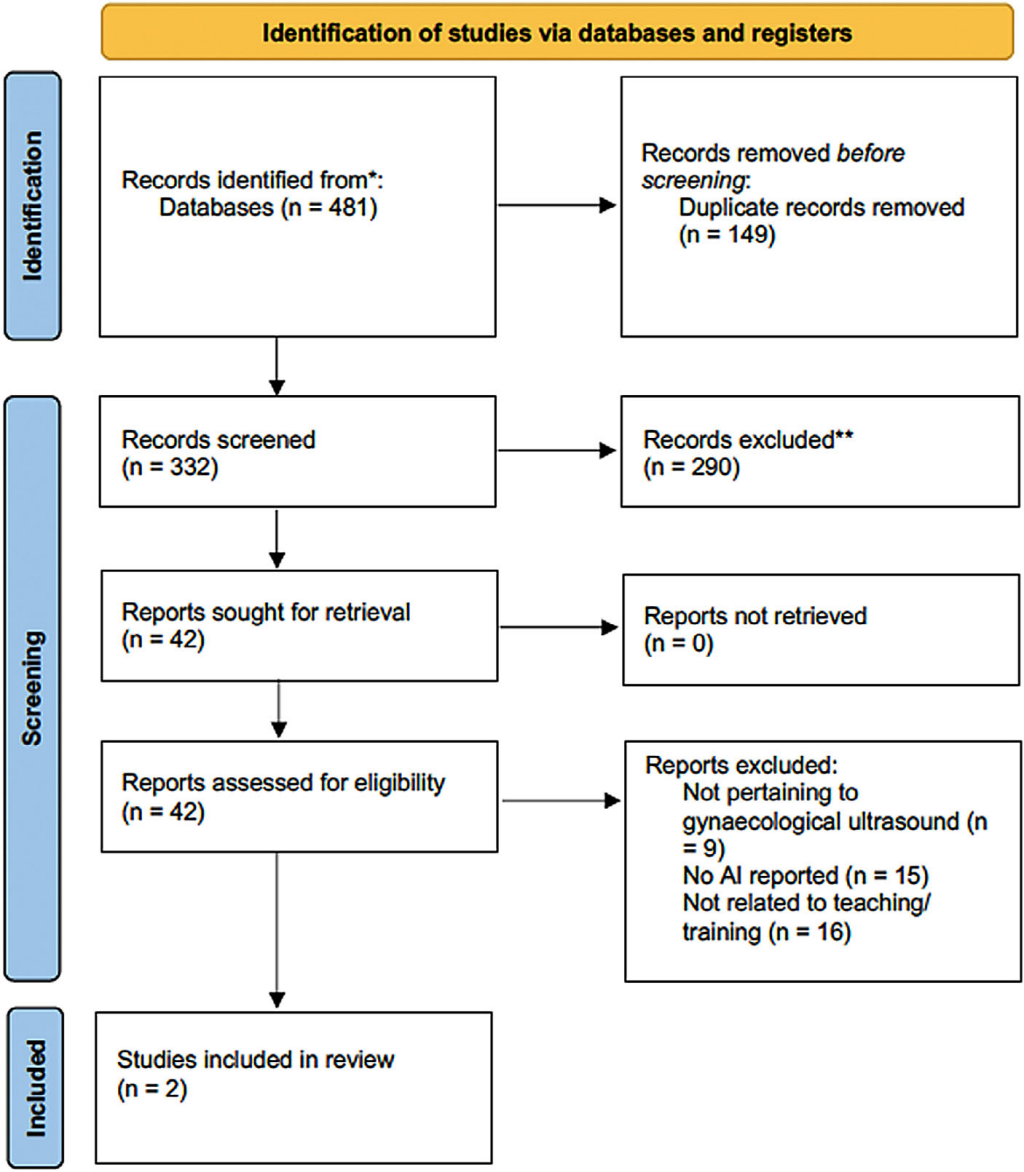
PRISMA flow diagram outlining the methods utilised for this systematic scoping review.

The search was performed on 15 December 2022. The initial database search returned 481 articles, 149 of which were duplicates. Of the 332 articles which underwent title and abstract screening, 42 proceeded to full‐text review and of these, two were deemed to meet the inclusion criteria.^2^, ^15^ Full details of the screening method can be seen in Figure 1.^13^

### Article characteristics

A summary of the included articles can be seen in Table 3. Neither of the articles included reported original research. Dhombres *et al*.^15^ performed a systematic review to determine the number and type of articles published in obstetrics and gynaecology (O & G) focused journals relating to AI technology. Meanwhile, Drukker *et al*.^2^ provided a holistic review of key AI concepts, as they relate to obstetric and gynaecological ultrasound for clinicians, and medical imaging more broadly. Both included articles that had been published within the last 3 years.

**Table 3:** Summary of the characterising and main findings of the included articles.

Author	Year of publication	Country	Article type	Aim	Results	Summary of key findings
Drukker *et al*.	2020	UK	Review Article	Provide an explanation of the key concepts relating to the use of AI in obstetric and gynaecological ultrasound for clinicians.	N/A	General review of the current utility of AI in obstetric and gynaecological ultrasound. This review did not reference any original research which had specifically been conducted into AI being used to teach or quality improve in gynaecological imaging yet reported three published papers published reporting AI being used for quality assurance of fetal imaging. A further three papers, pertaining to other applications of medical imaging, in which AI was used in navigation of the probe and identification of correct planes were discussed. This paper suggests that AI holds potential to assist in tasks such as identifying a good‐quality acquisition and providing real‐time (instant) quality assurance.
Dhombres *et al*.	2022	France	Systematic Review	Systematic review assessing the contribution of articles reporting AI in obstetrics and gynaecology focused journals.	A total of 66 papers were included, three of which specifically related to gynaecology and several relating to AI in ultrasound imaging in obstetrics or fetal medicine. One included paper covered the use of AI for ‘guided ultrasound image analysis’.	Currently, there is limited reporting of the use of AI for real‐time image analysis. This systematic review reported no specific studies on the use of AI for teaching of ultrasound skills. Citation of AI reporting articles are increasing annually, suggesting this is a growing field of research.

### Main findings

The main findings of the included articles can be seen in Table 3. Of the 66 articles Dhombres *et al*.^15^ included in their systematic review, three related to gynaecology (specifically oncology) and several referred to AI in obstetrics ultrasound imaging. One of their included papers covered the use of AI for ‘guided ultrasound image analysis’; however, further details of exactly what this involved were unclear. Furthermore, they reported that AI related articles in O & G focused journals typically reported the application of well‐established artificial neural networks (ANNs), such as U‐Net, being applied in a new context and that citation of such articles were increasing annually. Drukker *et al*.^2^ similarly reported a sharp increase in the inclusion of AI focused abstracts at O & G focused conferences in recent years. Within their review, Drukker *et al*.^2^ described three papers which reported AI being used for quality assurance of fetal imaging.^16^, ^17^, ^18^ A further three papers were included pertaining to other applications of medical ultrasound,^19^, ^20^, ^21^ such as echocardiography,^22^ in which AI was used in navigation of the probe and identification of correct scanning planes. They discussed how AI has the potential to do the same for gynaecological ultrasound.

## Discussion

This scoping review identified and evaluated published works related to the use of AI tools in the teaching of gynaecological ultrasound skills. Limited published data exists on this topic with no original research studies identified reporting any specific teaching tools which have been developed or which are currently under development. The articles included in this review discussed how AI is being currently used in gynaecological ultrasound, such as for the automation of measurements.^2^ Within wider literature, reports of tools performing automated measurements of the endometrium,^23^ levator hiatus^24^ and ovarian follicles^25^, ^26^ exist. As reported by Dhombres *et al*.,^15^ most AI articles relating to gynaecology, centre on oncology and the differentiation of benign from malignant ovarian lesions.^27^, ^28^, ^29^, ^30^ Christiansen *et al*.^30^ reported that a deep neural network image analysis had the ability to differentiate benign from malignant ovarian masses with comparable accuracy to expert human operators. In a recent systematic review and meta‐analysis, Xu *et al*.^31^ determined AI could perform as well as, or superior to human experts, further highlighting the prospect of AI as a clinical decision support system but noted that few studies have been externally validated. Until these techniques can be adequately validated *via* external validation, their clinical applicability is limited at this time and clinicians need to be cognisant of this. Both reviewed papers^2^, ^15^ spanned both O & G and therefore alluded to the concept of AI for the teaching of ultrasound scanning skills as a potential future application for gynaecological ultrasound, rather than a current reality. This assumption was based on similar applications having been developed and reported relating to obstetric ultrasound.

Obtaining the skills required to perform diagnostic gynaecological ultrasound is challenging. The skill necessary to perform at TVUS is typically acquired through a combination of theoretical study and hands‐on tuition with a skilled mentor until an adequate level of competence is achieved. Access to tuition with a mentor to upskill in the performance of TVUS is difficult due to its labour‐intensive nature, high cost and lack of experienced tutors in some locations. Providing direct supervision of a learner requires multiple people to be present in the ultrasound room.^32^ For an intimate examination like TVUS, this can be undesirable for patients. Having additional people present in ultrasound rooms is also a challenge which has been heightened in recent times due to COVID‐19 mitigation measures. Limited access to skilled tutors makes the teaching of TVUS skills impossible in some settings such as developing nations and remote locations due to the lack of experienced operators in such locations. According to the Australian Professional Competency Framework for Sonographers,^7^ endometriosis ultrasound, 3D/4D ultrasound or pelvic floor imaging are considered advanced/specialised skills rather than core skills. As such, many sonographers do not possess the skills to perform these scans. This results in a flow on effect, of having few people who possess the skills be able to teach these techniques adequately.^33^

Simulation education has been shown to be helpful in the teaching of ultrasound skills.^34^ High‐fidelity patient simulation units, in which an operator can manipulate a transducer on a mannequin whilst viewing the resulting scan on a computer, have been available in classrooms for several years.^34^, ^35^ Girzadas *et al*.^36^ found a combination of a transvaginal task trainer and high‐fidelity ultrasound mannequin improved the training experience of emergency medicine trainees learning TVUS. Similarly, Bahl *et al*.^37^ reported that the use of a sonographic simulator in the training of fertility nurses to perform ultrasound was helpful. Whilst helpful in teaching of ultrasound skills, such simulation training does not replace the need to scan real patients.^35^

In several (non‐gynaecological) facets of medical ultrasound, such as obstetric ultrasound and echocardiography, AI is beginning to be utilised as an adjunct to supervised scanning, to assist in the teaching of ultrasound skills, and image acquisition to inexperienced operators. Hartmann *et al*.^38^ described a system that allowed trainees to utilise a simulation app which allowed for practising of fetal heart ultrasound without the need for an ultrasound machine, patient or tutor to be present. Similar applications have been developed in the field of echocardiography^12^, ^39^ such as an app described by Langet *et al*.^39^ which guided novice users to obtain standardised views of the heart, giving real‐time feedback on the accuracy of the images, with prompts on how to improve. Such AI systems could have the potential to accelerate training of professionals using ultrasound whilst gaining real‐world experience by scanning real patients.

A strength of this review was the robust methodology, using best accepted methods for scoping reviews. The searching of both medical and computer science databases was also a strength of this study as we maximised the chances of finding works published in this area. Furthermore, the review team is a multidisciplinary group comprising experts in ultrasound, gynaecology, epidemiology, computer science and evidence review.

A limitation of this review was the little data reported in the included articles in relation to the research question. As this is a very novel and emerging field, the decision to not include conference abstracts may have reduced our chances of finding some preliminary studies which have been conducted in this area and is another limitation of this review. Given the potential for commercialisation of AI tools applied in this way, it is possible that some research exists which may be under embargo due to commercial implications.

## Conclusion

Artificial intelligence can potentially assist in the training of sonographers and other ultrasound operators, including in the field of gynaecological ultrasound. This scoping review revealed however that to date, no original research has been published reporting the use or development of such a tool specifically for gynaecological ultrasound. In obstetric ultrasound and echocardiography, AI is beginning to be utilised as an adjunct to supervised scanning, to assist in the teaching of ultrasound skills and there is a potential for this technology to be developed in the same way for gynaecological ultrasound in the future.

## Author contributions

All authors contributed to the conceptualisation, methodology, data analysis, writing and reviewing of this manuscript.

## Conflict of Interests

No authors have conflicts of interest to disclose in relation to this review.

## Supporting information


**Appendix S1.** The PRISMA‐ScR checklist.

**Appendix S2.** Logic grids outlining the keywords, synonyms and Boolean operators for each of the databases used in the search.
