# Supplementary material for: Artificial intelligence as a teaching tool for gynaecological ultrasound: A systematic search and scoping review
Source: Australas J Ultrasound Med. 2023 Nov 20;27(1):5–11. doi: 10.1002/ajum.12368 (PMC10902831; doi:10.1002/ajum.12368)
Supplement: Supplementary file 1 — Appendix S1. The PRISMA‐ScR checklist. [file AJUM-27-5-s001.pdf]

Appendix 2: Logic grids outlining the keywords, synonyms, and Boolean operators for each of the databases used in the search.

Medline

**AI tools**	**ultrasound**	**gynaecology**	**teaching**
Exp Artificial intelligence OR (artificial intelligence OR deep learning OR AI OR machine learning OR computer aided OR computer vision OR Neural Network).ti,ab	Ultrasonography.sh OR (ultraso* OR sonogr* OR POCUS).ti,ab	Gynecology.sh OR (gyn?ecolog* OR female pelvi*).ti,ab	Exp education OR (teach* OR train* OR instruct* OR upskill* OR learn* OR assist*).ti,ab

EMBASE/ EMCARE

**AI tools**	**ultrasound**	**gynaecology**	**teaching**
Exp Artificial intelligence OR (artificial intelligence OR deep learning OR AI OR machine learning OR computer aided OR computer vision OR Neural Network).ti,ab	Exp Echography OR transvaginal.sh OR (echography OR ultraso* OR sonogram* OR POCUS).ti,ab	Gynecology.sh OR (gyn?ecolog* OR female pelvi*).ti,ab	Exp education OR (teach* OR train* OR upskill* OR instruct* OR assist*)

CINAHL

AI tools	ultrasound	gynaecology	teaching
MH artificial intelligence+ OR TI AI OR AB AI OR TI “machine learning” OR AB “machine learning” OR TI “deep learning” OR AB “deep learning” OR TI “computer aided” OR AB “computer aided” OR TI “computer vision” OR AB “computer vision” OR TI “Neural Network” OR AB “Neural Network”	MH Ultrasonography+ OR TI ultraso* OR AB ultraso* OR TI sonogr* OR AB sonogr* OR TI POCUS OR AB POCUS	MH gynecology OR TI gynaecol* OR AB gynaecol* OR TI gynecol* OR AB gynecol* OR TI “female pelv*” OR AB “female pelv*”	MH education, health sciences+ OR TI teach* OR AB teach* OR TI train* OR AB train* OR TI instruct* OR AB instruct* OR TI upskill* OR AB upskill* OR TI assist* OR AB assist*

SCOPUS/ Web of Science

AI tools	ultrasound	gynaecology	teaching
{artificial intelligence} OR AI OR {machine learning} OR {deep learning} OR {computer aided} OR {computer vision} OR {Neural Network}	ultraso* OR sonogr* OR POCUS	gynaecol* OR gynecol* OR “female pelv*”	Teach* OR train* OR instruct* OR upskill* OR assist*

IEEE Xplore and ACM digital library

**AI tools**	**ultrasound**	**gynaecology**	**teaching**
“artificial intelligence” OR AI OR “machine learning” OR “deep learning” OR “computer aided” OR “computer vision” OR “Neural Network”	ultraso* OR sonogr* OR POCUS	gynaecol* OR gynecol* OR “female pelv*”	Teach* OR train* OR instruct* OR upskill* OR assist*
